# Clinicopathological features, risk and survival in lung cancer survivors with therapy-related acute myeloid leukaemia

**DOI:** 10.1186/s12885-020-07603-9

**Published:** 2020-11-10

**Authors:** Huabin Wang, Yin Yin, Ru Wang, Junbin Huang, Hongman Xue, Yucai Cheng, Lidan Zhang, Chun Chen

**Affiliations:** 1 Pediatric Blood Center, the Seventh Affiliated Hospital of Sun Yat-sen University, 628 Zhenyuan Road, Guangming, Shenzhen, Guangdong 518107 P.R. China; 2 Department of Thoracic Surgery, the Seventh Affiliated Hospital of Sun Yat-sen University, Shenzhen, Guangdong China; 3 Department of Radiation Oncology, Shandong Provincial ENT Hospital Affiliated to Shandong University, Jinan, Shandong China

**Keywords:** Therapy-related acute myeloid leukaemia, Lung cancer, Incidence risk, Survival analysis, SEER database

## Abstract

**Background:**

A secondary malignancy is the most serious complication in lung cancer (LC) survivors. This study aimed to evaluate the clinicopathological features, predictable risk factors and survival of patients with LC who developed therapy-related acute myeloid leukaemia (t-AML).

**Methods:**

Patients from the Surveillance, Epidemiology, and End Results (SEER) database diagnosed with t-AML after LC between 1975 and 2015 were included. Standardized incidence ratios (SIRs) were used to perform multiple primary analyses. The risk of t-AML development among LC patients was assessed using a logistic regression model. Kaplan–Meier analysis was used to construct overall survival (OS) curves. Cox regression was used to assess the influence of various prognostic factors.

**Results:**

A total of 104 patients with t-AML after LC-targeting chemotherapy were included. The median latency period to the development of t-AML was 35.5 months. The calculated SIR of t-AML was 4.00. Chemoradiotherapy, small cell lung cancer (SCLC), or localized/regional-stage LC was a risk factor for the development of t-AML. The median OS was only 1 month, and those younger than 65 years were predicted to have a better OS time.

**Conclusions:**

t-AML is a rare but serious late complication in LC patients and is associated with a poor prognosis. It is necessary to carry out long-term follow-up and screen for t-AML in LC patients, especially among those undergoing both radiotherapy and chemotherapy, with SCLC or with localized/regional-stage LC.

## Background

Among cancers, lung cancer (LC) has the highest incidence and mortality rates worldwide. The estimated number of new cases of LC in the United States in 2019 was 228,150, and the estimated number of deaths was 142,670 [1]. The poor prognosis of LC is related to many factors, among which the occurrence of a secondary malignant tumour is a great obstacle for a disease-free status.

The development of therapy-related acute myeloid leukaemia (t-AML) has always been considered a rare but highly serious complication of cytotoxic chemotherapy and radiotherapy [2]. It is estimated that t-AML after LC accounts for 5.9% of all t-AML and is the third most common malignant tumour after breast cancer and non-Hodgkin’s lymphoma [3]. Recently, an increase in t-AML incidence associated with increased use of adjuvant cancer treatment and a prolonged life span of cancer survivors has been reported [4, 5]. However, there are only scattered reports regarding t-AML after LC [6–11], and no study has systematically investigated its clinical characteristics, risk and survival. In general, it is crucial to identify high-risk groups to help with individualized treatment and further improve the prognosis of patients with LC.

The purpose of this study was to identify the clinicopathological features of LC-related t-AML and predictable risk factors and survival of patients with LC who developed t-AML by analysing the Surveillance, Epidemiology, and End Results (SEER) database. The findings will offer treatment guidance for physicians.

## Methods

### Data source

In this retrospective population-based study, information for patients diagnosed with t-AML after treatment for primary LC was obtained from the SEER-9 database. The SEER database is a system of cancer registries that incorporates high-quality data. In the present study, we included 9 cancer registries (namely, Atlanta, Connecticut, Detroit, Hawaii, Iowa, New Mexico, San Francisco-Oakland, Seattle-Puget Sound, and Utah) as released in November 2018. This cohort covers approximately 10% of the total US population. According to the limited-use protocol of SEER research data, these data can be used publicly, and approved by the institutional review committee was not required for our research.

### Inclusion criteria, exclusion criteria and data extraction

Subjects diagnosed with t-AML after a primary LC diagnosis were identified from the multiple primary-standardized incidence ratio (SIR) method in SEER*Stat (version 8.3.6). From 1975 to 2015, we first screened patients whose malignant tumours were located in the lung by using the selected function of multiple primary SIRs. All of these patients with LC had received chemotherapy during LC treatment. From this group, we further selected patients who developed t-AML after LC diagnosis during this period. The following criteria were used to exclude patients from this study: (1) age less than 20 years; (2) history of malignancy in addition to LC; (3) diagnosis of AML within 2 months after LC diagnosis; and (4) incomplete survival information.

Demographic parameters were extracted, including sex, age, ethnicity, and the period of diagnosis, as were histopathological LC data, including stage, pathologic grade and histologic type. The SEER historical stage was used for LC staging [12, 13]. It categorizes cancer cases as localized, regional, distant, or unknown based on the following definitions: the localized stage indicates that the neoplasm is confined entirely to the organ of origin; the regional stage includes regional disease by direct extension or lymph node involvement; and the distant stage indicates that the neoplasm spreads to parts of the body distant from the primary tumour. Pathological grade was divided into four grades: grade I, well differentiated; grade II, moderately differentiated; grade III, poorly differentiated; grade IV, undifferentiated. Histologic types include small cell lung cancer (SCLC) and non-small cell lung cancer (NSCLC). Treatments for LC were recorded including the radiotherapy status and surgery status. The latency period (LP) was defined as the interval from the diagnosis of LC to the diagnosis of t-AML. We recorded whether the patient was alive or deceased, and overall survival (OS) was calculated as the number of months starting from the t-AML diagnosis to the death of the patient or last follow-up recorded (December 31, 2015).

### Statistical methods

SIRs, excess risk (ER) and person-years at risk were chosen for primary analyses, as generated from the multiple primary-SIR analysis. SIRs were used to compare the incidence of AML in primary LC patients who had received chemotherapy after LC diagnosis to the incidence of AML in the general population. We also computed the ER to estimate the number of excess t-AML patients per 10,000 persons per year.

Categorical data were compared using the chi-square test, and the results are expressed as proportions. Odds ratios (ORs) of t-AML development risk in patients with LC were obtained using a logistic regression model. OS curves were established by Kaplan–Meier analysis and compared using the log-rank test. Finally, Cox regression was employed to evaluate the effect of various prognostic factors on OS.

Variables that had a *P* value < 0.20 in univariate analysis were included in the multivariate logistic or Cox regression model. Multicollinearity was tested using the variance inflation factor (VIF) method and, a VIF ≥5 was not permitted. The statistical analysis was performed using “R” version 3.6.2 (http://www.r-project.org) and SPSS 24.0 (IBM-SPSS, Armonk, NY). All *P* values were two-sided and considered statistically significant at < 0.05.

## Results

### Patient demographics

One hundred and four patients with t-AML after LC diagnosis and 158,541 patients with primary LC who did not develop t-AML between 1975 and 2015 in the SEER-9 database were included. Table 1 describes the demographic variables for these patients. Half of the patients with t-AML were men (50%). No significant difference was found between LC patients with t-AML and LC patients without t-AML in terms of the median age at LC diagnosis (62.5 years vs. 64 years, respectively; *P* = 0.332). Over time, the number of LC patients with or without t-AML increased to varying degrees, especially those with t-AML, with a sevenfold increase. The proportion of LC patients who developed t-AML was higher in those who underwent surgery (*P* = 0.006) or radiotherapy (*P* = 0.001) during LC treatment and in those with localized/regional-stage LC (*P* < 0.001) than in those who did not.

**Table 1:** Demographic characteristics of primary LC patients with or without t-AML

Variables, No. (%)	With t-AML (***N*** = 104)	Without t-AML (***N*** = 158,541)	P
**Sex**			0.087
Male	52 (50)	92,390 (58.3)	
Female	52 (50)	66,151 (41.7)	
**Race**			0.999
White	86 (82.7)	130,695 (82.4)	
Black	11 (10.6)	16,891 (10.7)	
Other	7 (6.7)	10,824 (6.8)	
**Age of LC diagnosis, median (range)**	62.5 (41–82)	64 (10–100)	0.332
< 65 years	58 (55.8)	80,876 (51.0)	
≥ 65 years	46 (44.2)	77,665 (49.0)	
**Period of diagnosis**			0.131
1975–1984	7 (6.7)	22,500 (14.2)	
1985–1994	12 (11.5)	33,044 (20.8)	
1995–2004	29 (27.9)	45,654 (28.8)	
2005–2015	56 (53.8)	57,342 (36.2)	
**Surgical treatment**			0.006
Yes	22 (21.2)	19,395 (12.2)	
No	82 (78.8)	139,146 (87.8)	
**Radiation treatment**			0.001
Yes	78 (75.0)	92,389 (58.3)	
No/unknown	26 (25.0)	66,152 (41.7)	
**Stage**			< 0.001
Localized/regional	53 (51.0)	41,086 (25.9)	
Distant	39 (37.5)	81,744 (52.2)	
Unknown	12 (11.5)	34,711 (21.9)	
**Pathologic grade**			0.659
I or II	13 (12.5)	16,116 (10.2)	
III or IV	39 (37.5)	64,510 (40.7)	
Unknown	52 (50.0)	77,915 (49.1)	
**Histologic type**			0.09
SCLC	42 (40.4)	51,078 (32.2)	
NSCLC	59 (56.7)	96,664 (61.0)	
Unknown	3 (2.9)	10,799 (6.8)	

*LC* Lung cancer, *t-AML* Therapy-related acute myeloid leukaemia, *NSCLC* Non-small cell lung cancer, *SCLC* Small cell lung cancer, *SEER* Surveillance, Epidemiology, and End Results

### Risk of t-AML

#### Comparison with the general population

The calculated SIR of t-AML was 4.00 (95% CI: 3.28–4.82), with an ER of 3.66 cases per 10,000 persons. Regarding patients with t-AML, SIRs were increased to various degrees for different LPs, calendar periods and ages at diagnosis (Table 2), and the median LP for the t-AML patient cohort was 35.5 months (range: 3–255 months). High SIRs were concentrated in 1–5 years, especially in the 3–5 years after LC diagnosis (SIR: 9.29; 95% CI: 6.35–13.11). The SIR was 5.99 (95% CI: 2.41–12.35) for the 1975–1984 period, and that for patients aged < 65 years was 9.08 (95% CI: 6.74–11.97).

**Table 2:** Standardized incidence ratios and excess risk for the diagnosis of t-AML after lung cancer

Characteristic	n (%)	SIR (95% CI)	ER (per 10,000)	Person-years at risk
**Sex**				
Male	52 (50.0)	3.20 (2.41–4.16)^*^	3.26	116,136.88
Female	52 (50.0)	5.36 (4.03–7.00)^*^	4.10	107,177.52
**Age**				
< 65 years	58 (55.8)	9.08 (6.74–11.97)^*^	4.24	104,996.47
≥ 65 years	46 (44.2)	2.71 (2.07–3.50)^*^	3.15	118,317.93
**Latency period**				
2–11 months	11 (10.6)	1.12 (0.56–2.00)	0.13	86,808.04
12–35 months	41 (39.4)	5.35 (3.87–7.21)^*^	5.15	67,848.48
36–59 months	31 (29.8)	9.29 (6.35–13.11)^*^	10.54	27,090.39
60+ months	21 (20.2)	3.88 (2.46–5.82)^*^	4.11	41,567.48
**Period of diagnosis**				
1975–1984	7 (6.7)	5.99 (2.41–12.35)^*^	4.26	13,687.57
1985–1994	12 (11.5)	4.19 (2.23–7.17)^*^	3.01	32,838.60
1995–2004	29 (27.9)	4.05 (2.73–5.78)^*^	3.61	62,575.81
2005–2015	56 (53.8)	3.79 (2.88–4.88)^*^	3.80	114,212.42

*ER* Excess risk, *t-AML* Therapy-related acute myeloid leukaemia, *SIRs* Standardized incidence ratios

^*^ Indicates *P* < 0.05

#### Internal comparisons among cohorts

**Fig. 1:**
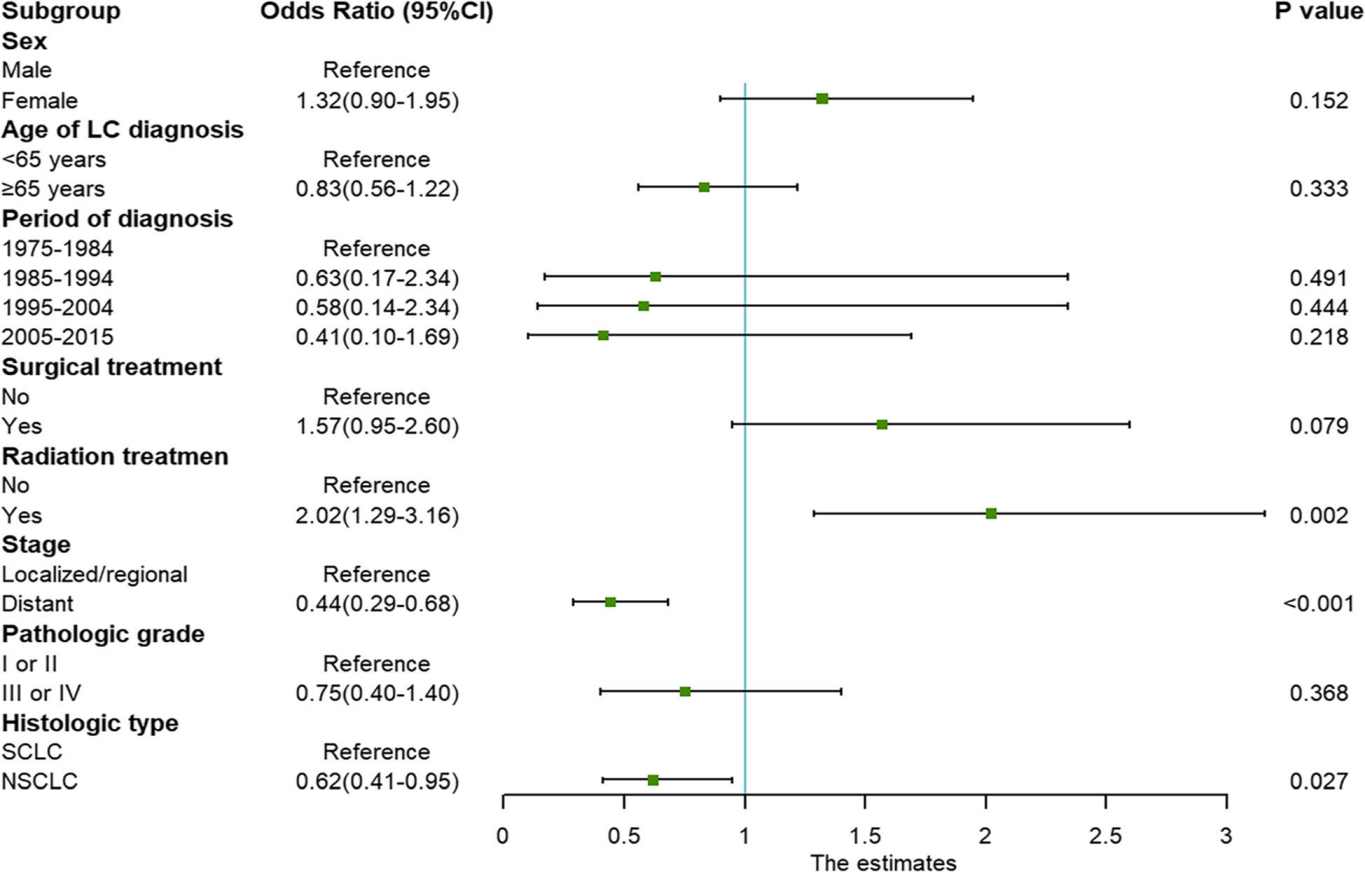
Multivariate logistic regression model exploring risk factors for developing t-AML among LC patients

The calculated incidence of t-AML after chemotherapy for LC was 0.07%. To identify risk factors for t-AML in the primary LC population, we compared t-AML patients with 158,541 primary LC patients, and the impacts of various patient characteristics on the risk of t-AML development were analysed using logistic regression. Radiotherapy combined with chemotherapy, SCLC and localized/regional-stage LC were the main risk factors for t-AML development (Fig. 1). Other factors, including age, sex, the period of LC diagnosis, pathologic grade and surgery, did not reach statistical significance.

### Survival of patients with t-AML

**Fig. 2:**
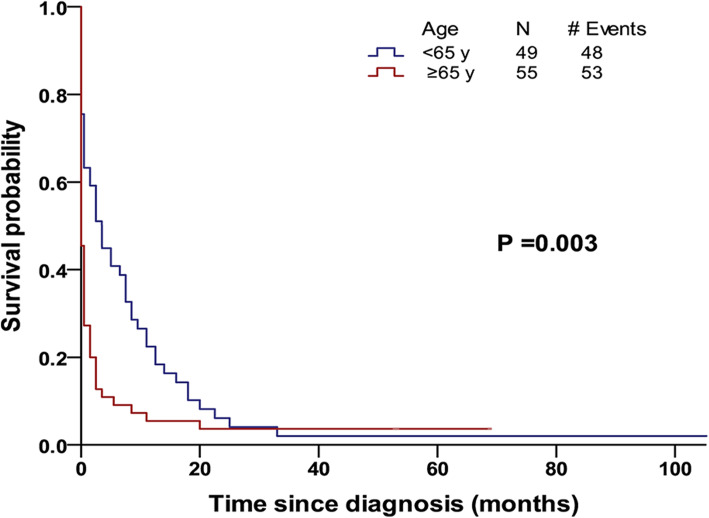
Kaplan–Meier plots depicting overall survival for t-AML patients based on age

**Fig. 3:**
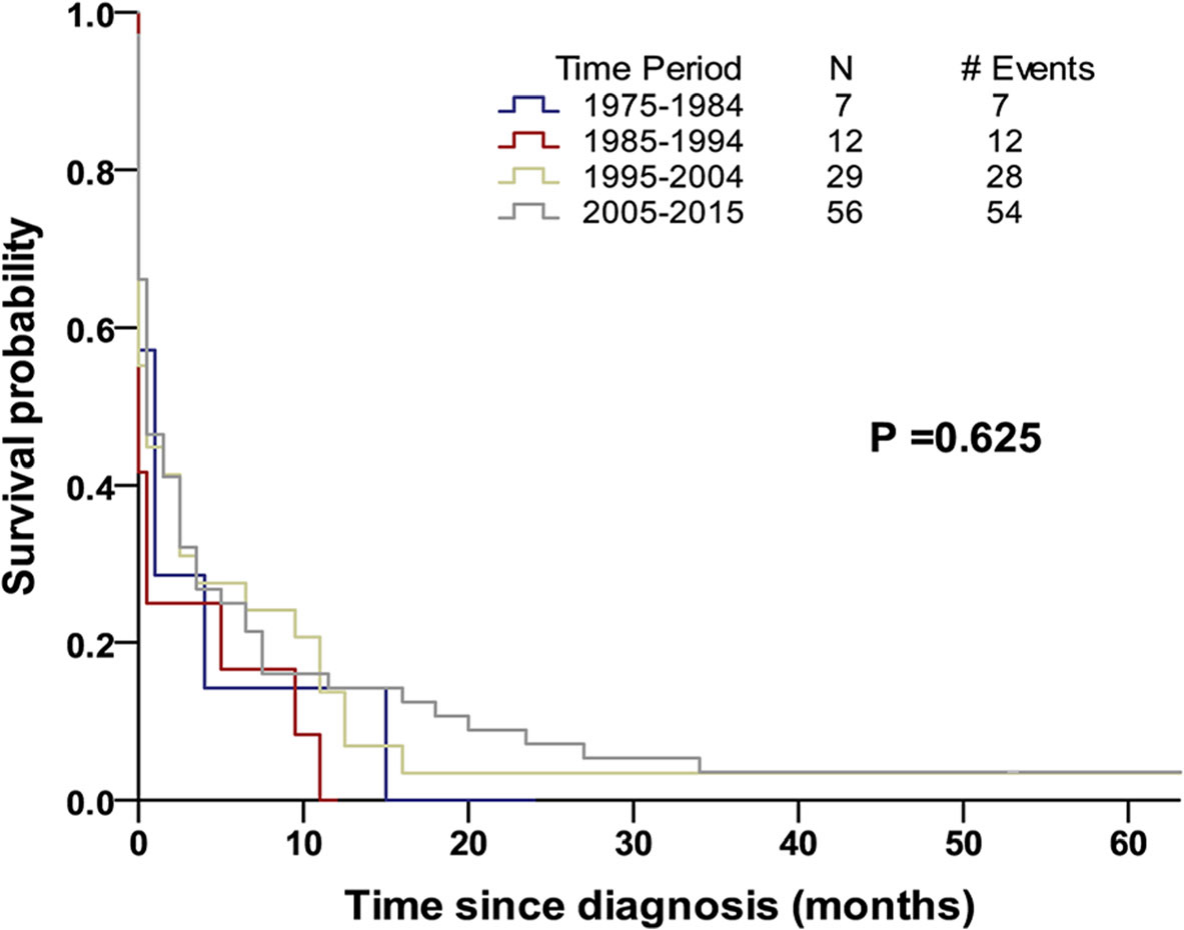
Kaplan–Meier plots depicting overall survival based on the period of diagnosis for patients with t-AML

The median follow-up time of patients with t-AML was 37.8 months (range: 2–257 months). In total, 97.1% of the patients died before the end for this study. The median OS was only 1 month (95% CI: 0.4–1.6) (Table 3), and the 6-month, 1-year, 2-year and 5-year OS rates were 25, 13, 6, and 3%, respectively. In the t-AML cohort, 42 patients (40.4%) had a survival time of less than 1 month, and most of them were aged ≥65 years (30 patients). Further analyses based on age stratification at the time of diagnosis indicated that patients who were diagnosed at an age < 65 years had a median OS of 4 months, whereas those who were diagnosed at an age ≥ 65 years had a median OS < 1 month (*P* = 0.003) (Fig. 2). Moreover, the 6-month and 1-year OS rates of patients diagnosed at an age < 65 years were higher than those of patients diagnosed at an age ≥ 65 years (6-month OS: 41% vs. 11%, respectively, *P* < 0.001; 1-year OS: 22% vs. 5%, respectively, *P* < 0.001). Nonetheless, the 2-year and 5-year OS rates were very low in both groups, with no statistically significant difference (2-year OS: 6% vs. 5%, respectively, *P* = 0.752; 5-year OS: 2% vs. 4%, respectively, *P* = 0.397). The median OS of patients with t-AML based on stratification by the period of diagnosis (i.e., 1975–1984, 1985–1994, 1995–2004 and 2005–2015) was not increased (2 months, 0 months, 1 month and 1 month, respectively) (*P* = 0.625) (Fig. 3).

**Table 3:** Survival of patients with t-AML based on age or period of diagnosis

	Median OS	*P* value ^a^	6-month OS	1-year OS	2-year OS	5-year OS
t-AML	1 month		25%	13%	6%	3%
**Age**		0.003				
< 65 years	4 months		41%	22%	6%	2%
≥ 65 years	< 1 month		11%	5%	5%	4%
**Period of diagnosis**		0.625				
1975–1984	2 months		14%	14%	0%	0%
1985–1994	< 1 month		25%	0%	0%	0%
1995–2004	1 month		28%	14%	3%	3%
2005–2015	1 month		25%	16%	9%	4%

*LC* Lung cancer, *t-AML* Therapy-related acute myeloid leukaemia, *OS* Overall survival

^a^The *P* value is the median OS of t-AML patients based on age or period of diagnosis

### OS prognostic variables

Multivariable analysis of the 104 patients with t-AML showed that only age < 65 years had predictive value for a longer OS (hazard ratio (HR): 0.59; 95% CI: 0.40–0.89; *P* = 0.012). However, patients with t-AML caused by localized/regional LC or who were recently diagnosed with LC during the 2005–2015 period did not have a longer OS. Other factors, such as sex, LP, pathologic grade, surgery, radiation and pathological type of LC, showed no predictive value for OS.

## Discussion

The risk of developing t-AML in survivors of LC and their survival conditions are increasingly raising concern because the use of active modern therapies and the number of survivors is drastically growing. Our research used the latest data provided by the SEER database to evaluate clinicopathological features, risk and survival among patients with t-AML after LC. To the best of our knowledge, this information has never been previously reported. According to our results, among the different age, LP, period of diagnosis and sex groups, patients with t-AML after LC had higher SIRs. Multivariable analysis showed that within the LC patient group, pathological classification of LC, stage and radiotherapy were indicators of t-AML development. Finally, patients with t-AML had a very poor prognosis, with a median OS of only 1 month, and only age < 65 years was a factor for a longer OS. No difference in median OS was found in other subgroups, including LC stage and period of diagnosis subgroups.

Eighty percent of all patients with t-AML were diagnosed within the first 5 years after their LC diagnosis; the median LP from LC diagnosis to t-AML diagnosis was 35.5 months. The other 20% of patients with t-AML were diagnosed more than 5 years after their LC diagnosis, emphasizing the necessity of long-term follow-up for patients with LC. The most common cause of t-AML is exposure to alkylating agents or radiotherapy, usually after an LP of approximately 5–7 years [14], and patients with t-AML due to exposure to topoisomerase II inhibitors have a relatively shorter LP of 1–3 years [14]. We speculate that the rapid progression of t-AML in our cohort may be related to the use of various cytotoxic chemotherapies. As first-line chemotherapy drugs of NSCLC, some anti-tumour drugs from plants, including paclitaxel, are reported to have an LP of only 1–2 years [15]. Regardless, this research was conducted on a small-sized sample, leading to limited statistical credibility. The poor prognosis of patients with LC may also lead to a shorter LP. Additionally, the median age of t-AML diagnosis in a previous study was 61 years or younger [5, 16, 17], whereas the median age of patients with t-AML in our study was 65 years. This may be explained by an older age at primary LC diagnosis than for other cancer types.

Except within the first year after LC diagnosis, patients with LC had higher SIRs of developing t-AML than the general population, regardless of their sex, age, LP and diagnosis period. This emphasizes the universality of high risk of t-AML development in LC patients. In the primary LC cohort, multivariate analysis showed that those who received both chemotherapy and radiotherapy had an increased risk of developing t-AML compared with those who received chemotherapy alone. This finding was consistent with previous reports [2, 18]. Nevertheless, radiotherapy as a t-AML risk factor remains controversial. For example, Agnieszka et al. [19] investigated 109 patients with t-AML after treatment for epithelial ovarian carcinoma in a population-based study, and their results showed that a combination of chemotherapy and radiotherapy did not increase the risk of developing t-AML compared with chemotherapy alone (HR: 1.45; 95% CI: 0.71–2.96).

Additionally, the pathological type and stage of LC were found to be important factors affecting the occurrence of t-AML. The cumulative risk of t-MDS/t-AML after intensive treatment targeting SCLC has been reported to be 14% at 4 years after diagnosis [20] and 25% at 3 years after diagnosis [21]. Unfortunately, neither of these studies compared NSCLC with SCLC. Our study showed for the first time that SCLC was more likely to lead to t-AML development than NSCLC. First, SCLC is more sensitive to radiotherapy and chemotherapy than NSCLC; therefore, patients with the former are more likely to receive longer chemotherapy courses and higher doses of chemotherapy drugs than patients with the latter. Second, the topoisomerase inhibitor II etoposide is the first-line chemotherapy for SCLC, and its toxicity to bone marrow is higher than that of conventional third-generation chemotherapy drugs for NSCLC [22]. Stage as another risk factor suggests that patients with highly aggressive LC did not survive long enough to develop t-AML. Furthermore, patients who survive longer are more likely to receive multiple courses of chemotherapy in their lifetime.

Our observations did not reveal difference in the risk of developing t-AML after LC during different calendar periods, in contrast to what has been previously reported [4, 19]. In one study, the general improvement of the chemotherapy dosage and toxicity in recent years was found to have reduced the risk of t-AML development after primary cancer treatments [19]. In the 1975–1984 period, multiple drug combination schemes such as MACC and CAMP with an alkylating agent were recommended as the main regimens for treating LC [22]. In contrast, the main chemotherapy regimens in 1985–1994 and 1995–2015 were platinum combined with second-generation chemotherapy drugs such as vinblastine, cyclophosphamide, and mitomycin and third-generation chemotherapy drugs such as paclitaxel, vinorelbine, and docetaxel. The toxicity of modern third-generation chemotherapy is lower than that of second-generation chemotherapy. However, we did not observe a significant decrease in OR in the last period based on multivariate analysis. The SIRs of 1975–1984 appeared to be higher than those of the other calendar periods, and a larger sample size and long-term follow-up are needed to better assess the risk of the calendar period of t-AML development in LC patients undergoing current treatments, including immunotherapy and molecular targeted drugs.

We found that patients with t-AML had a very poor prognosis, with a median OS of only 1 month. With the advancement of supportive therapy over the past 40 years, the application of new chemotherapy drugs, such as CPX-351 [23], and the development of nonmyeloablative conditioning therapy as part of allogeneic haematopoietic cell transplantation have steadily improved the survival rate and quality of life of t-AML patients. Under the current treatment, the median OS of all t-AML patients is between 6 and 10 months [5, 24]. The survival rate of patients with advanced NSCLC has also increased significantly, with a median OS doubling time from 7 to 12–13 months [22]. However, similar improvement was not observed in patients with t-AML after LC. Indeed, we only observed a slightly increased median OS in patients with LC aged < 65 years. Another important finding was that patients aged < 65 years had only a short-term cumulative survival advantage in the first 2 years; the survival advantage no longer existed when more than 2 years has occurred since the t-AML diagnosis.

Several factors can potentially explain the poor outcome of t-AML. First, the survival rate of primary LC is already relatively low. Therefore, such leukaemias will further reduce the possibility of long-term survival in LC. Second, patients with t-AML are usually older, leading to weaker immunity and more age-related complications. Third, previous chemotherapy for LC may result in the depletion of haematopoiesis reserves, causing a longer bone marrow (BM) suppression time and more serious treatment-related complications after t-AML treatment. Finally, molecular and cytogenetic abnormalities associated with conventional chemotherapy resistance, such as TP53 mutations, are common in patients with t-AML, leading to a poor prognosis [25, 26]. Although clinicians always attribute a poor prognosis to poor cytogenetic abnormalities, it is evident that t-AML is an independent adverse prognostic factor unrelated to karyotype [16, 27].

An unanticipated finding was that only age younger than 65 years predicted improved OS in patients with t-AML. Several studies have shown that older age indicates a worse OS among patients with t-AML [16, 28]. This study found no other factors, such as the period of diagnosis or stage of LC, to be predictive of OS. This finding further highlights the poor prognosis of patients with t-AML from another aspect as well as little progress in the treatment of t-AML after LC.

There are several limitations in our study. First, four patients developed AML 10 years after the diagnosis of LC. Due to the lack of data, including cytogenetics and molecular genetics, in the SEER database, there is no way to accurately determine whether the occurrence of AML is due to chemotherapy/radiotherapy or is de novo AML. Moreover, these genetic characteristics may affect the prognosis of patients with t-AML. Second, in 1999, the World Health Organization lowered the threshold of BM blast percentage required for AML diagnosis from 30 to 20%. However, it is still debated whether the natural history and responsiveness to therapy of patients with 20–30% BM blast is comparable to that of patients with > 30% BM blast AML, and this may affect the analysis of t-AML in different periods. Third, the SEER database does not include all initial chemotherapy and radiotherapy information because some treatments may have been performed outside the hospital or medical centre that reported the case to the registration centre. Thus, the conclusion of this study may not represent all patients with LC receiving chemotherapy and radiotherapy. Fourth, no information about the type, dose and course of chemotherapy drugs in the SEER database is available, and information regarding whether patients with t-AML received palliative treatment or active treatment and whether haematopoietic stem cell transplantation was used is not provided. These factors may affect the analysis of high-risk factors and prognostic factors of t-AML.

## Conclusions

In conclusion, t-AML is a rare but serious late complication of patients with LC and has a poor prognosis. The conclusions of our study are valuable when explaining the risk of developing t-AML among patients with LC. The benefit of chemoradiotherapy and risk of developing t-AML should be weighed by physicians when selecting chemoradiotherapy regimens. It is necessary to carry out long-term follow-up and screening of t-AML among patients with LC, especially those receiving both radiotherapy and chemotherapy, with localized/regional-stage disease or SCLC. Furthermore, a prospective, multicentre study is urgently needed to improve the prognosis of patients with t-AML after LC. Additionally, uploading and sharing more patient data from various medical centres around the world would contribute to international cooperation to increase our understanding of such patients and find better treatment methods.

## Data Availability

The datasets analysed during the current study are available in the SEER*Stat software (version 8.3.6, download from https://seer.cancer.gov/data/options.html). A registration form needs to be completed before using the software, and filter criteria need to be added.

## Abbreviations

BM: Bone marrow
ER: Excess risk
HR: Hazard ratio
LC: Lung cancer
t-AML: Therapy-related acute myeloid leukaemia
LP: Latency period
NSCLC: Non-small cell lung cancer
OR: Odds ratio
OS: Overall survival
SCLC: Small cell lung cancer
SEER: Surveillance, Epidemiology, and End Results
SIR: Standardized incidence ratio
